# Supplementary figures and images for: Genetics of height and risk of atrial fibrillation: A Mendelian randomization study
Source: PLoS Med. 2020 Oct 8;17(10):e1003288. doi: 10.1371/journal.pmed.1003288 (PMC7544133; doi:10.1371/journal.pmed.1003288)

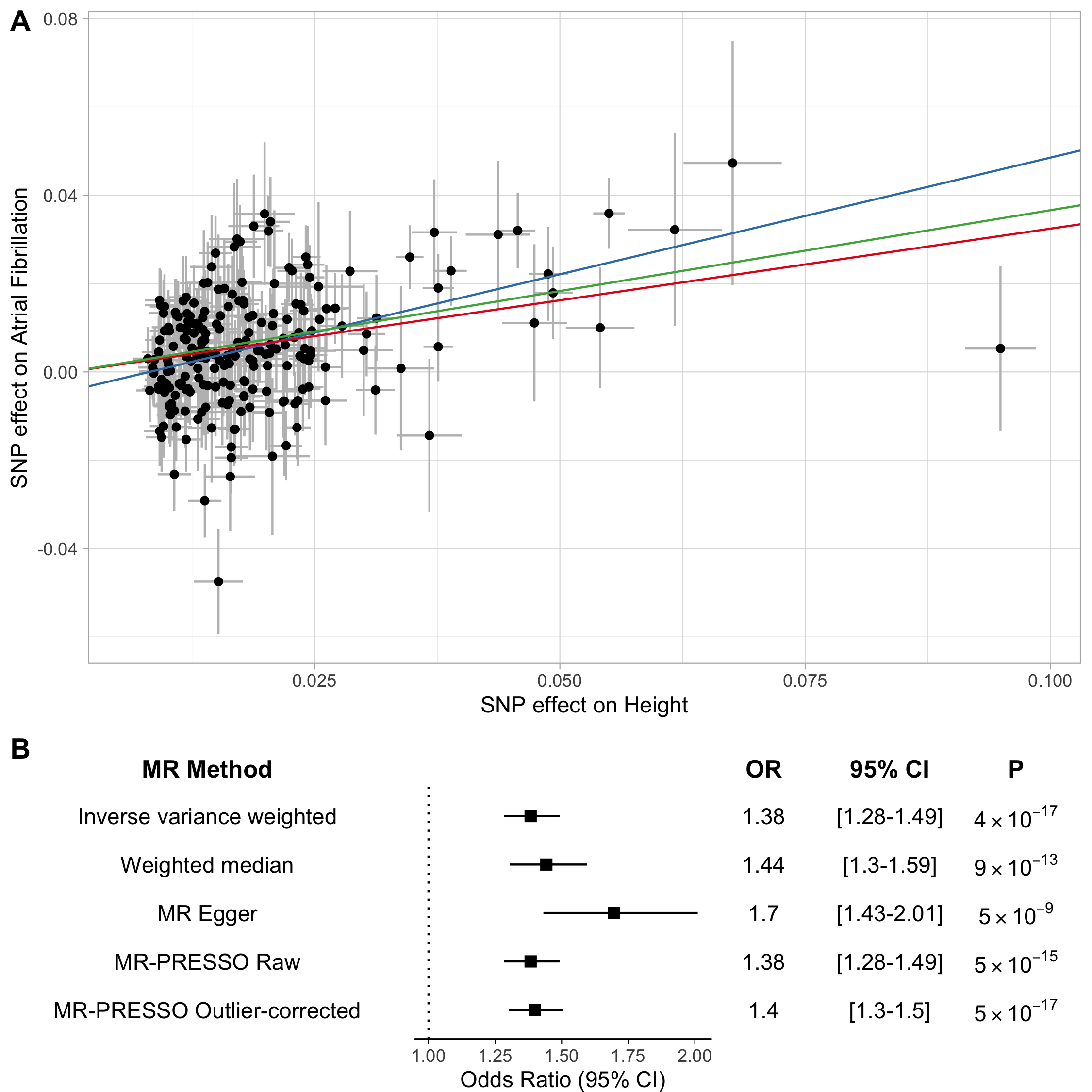

Supplement: S1 Fig — Two-sample MR was performed using a genetic instrument containing 224 independent SNPs associated with height, excluding SNPs nominally associated (p < 0.05) with traditional atrial fibrillation risk factors: coronary artery disease, HDL, LDL, total cholesterol, triglycerides, fasting glucose, fasting insulin, diabetes, BMI, waist-to-hip ratio, and systolic blood pressure. (A) Each point represents the SNP effects on height and atrial fibrillation. Colored lines represent inverse-variance-weighted (red), weighted median (green), and MR-Egger (blue) estimates of the association between a 1-SD increase in height and risk of atrial fibrillation. (B) Odds ratios (ORs), 95% confidence intervals (CIs), and p-values for MR estimates. (TIF) [file pmed.1003288.s001.tif]

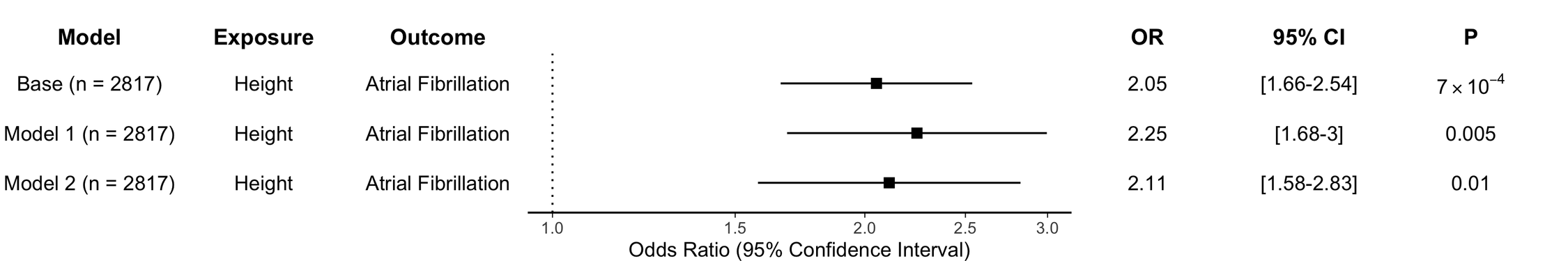

Supplement: S3 Fig — Individual-level instrumental variable analysis was performed in the subset of Penn Medicine Biobank participants with clinically obtained echocardiogram data, using a GRS for height as an instrumental variable for measured height. The base model was adjusted for age, sex, and 6 genetic principal components. Model 1 was additionally adjusted for weight, hypertension, coronary artery disease, heart failure, hyperlipidemia, diabetes, chronic kidney disease, sleep apnea, stroke, thyroid disease, smoking, cardiac surgery, and valvular heart disease. Model 2 was additionally adjusted for left atrial size as measured on transthoracic echocardiogram. Odds ratios (ORs) are reported per 1-SD increase in height. (TIF) [file pmed.1003288.s003.tif]

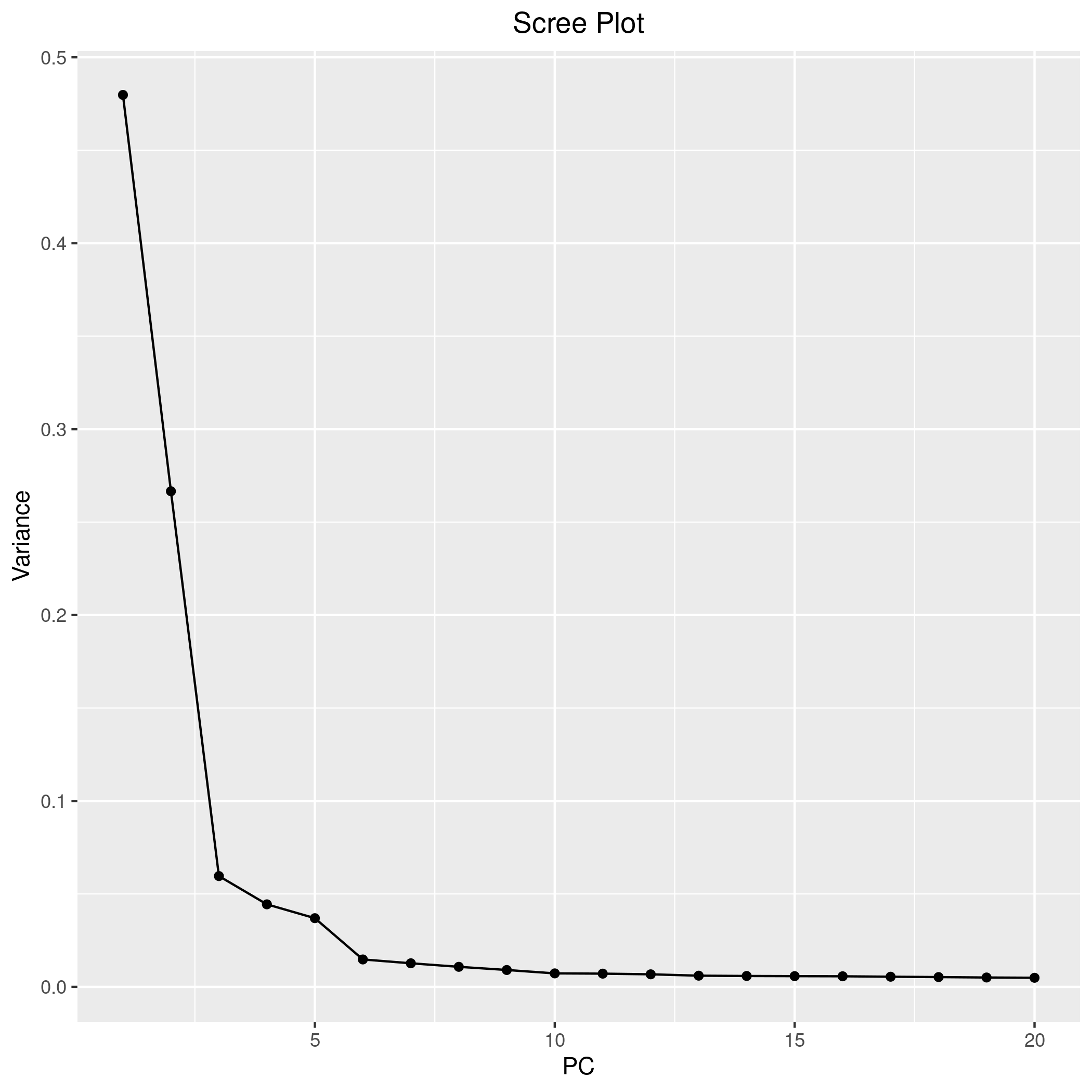

Supplement: S4 Fig — Proportion of variance explained for each genetic principal component among European-ancestry participants of Penn Medicine Biobank. (TIF) [file pmed.1003288.s004.tif]
