# Supplementary material for: Genetics of height and risk of atrial fibrillation: A Mendelian randomization study
Source: PLoS Med. 2020 Oct 8;17(10):e1003288. doi: 10.1371/journal.pmed.1003288 (PMC7544133; doi:10.1371/journal.pmed.1003288)
Supplement: S1 Methods — (DOCX) [file pmed.1003288.s005.docx]

**Genetics of Height and Risk of Atrial Fibrillation: A Mendelian Randomization Study**

**SUPPLEMENTAL METHODS**

**MENDELIAN RANDOMIZATION BIAS ESTIMATION**

The results of Mendelian randomization analyses may be biased, although the effects may differ depending upon the underlying analysis (single- vs. two-sample), type of outcome (binary vs. continuous), and sample overlap. The effects of weak instrument bias are well-characterized in the analysis by Burgess, Davies, and Thompson [1]. Weak instrument bias in the single-sample setting tends to bias estimates toward the observational estimates, while in the two-sample setting estimates are biased towards the null. With a binary outcome, estimating the instrument-exposure relationship in control participants only results in negligible bias, regardless of sample overlap. When the instrument-exposure association is estimated in both cases and controls, however, substantial bias may result. Two-sample MR often relies on effect estimates derived from large consortia, with instrument-exposure associations estimated in both cases and controls for the outcome of interest. Sample overlap may therefore lead to bias in the MR estimates, even in the case of two-sample MR. In the current study, a substantial proportion of UK Biobank participants were included in the genome-wide association studies of height and atrial fibrillation, which could potentially lead to biased estimates of the effect of height and atrial fibrillation. To estimate the potential bias due to varying proportions sample overlap, we used the calculator available at <https://sb452.shinyapps.io/overlap/>. For a range of sample overlaps (0 to 100%) we estimated the potential bias of the 707-variant genetic instrument explaining 11.2% of the variation in height, among 693,529 individuals in the GIANT + UKB height GWAS, and 588,190 individuals in the Roselli et. al. 2018 AFGen atrial fibrillation GWAS, with case-proportion of 0.11, and observational log-odds estimate of the effect of 1 standard deviation increase in height on risk of atrial fibrillation of 0.408 based on the observational pheWAS analysis in the Penn Medicine Biobank. Across the entire range of possible sample overlap we detected no substantial bias. For reference, the GIANT + UKB GWAS of height included 456,426 participant from the UK Biobank, while the AFGen GWAS of atrial fibrillation included 351,017. UK Biobank participants.

| **Overlap Proportion** | **Bias** | **Type I error rate** |
| --- | --- | --- |
| 0 | 0 | 0.05 |
| 0.1 | 0 | 0.05 |
| 0.2 | 0.001 | 0.05 |
| 0.3 | 0.001 | 0.05 |
| 0.4 | 0.001 | 0.05 |
| 0.5 | 0.002 | 0.05 |
| 0.6 | 0.002 | 0.05 |
| 0.7 | 0.002 | 0.05 |
| 0.8 | 0.003 | 0.06 |
| 0.9 | 0.003 | 0.06 |
| 1 | 0.003 | 0.06 |

## Penn Medicine Biobank Genotyping, QC and Imputation

DNA extracted from blood was genotyped on the Illumina OmniExpress or Global Screening Array chips. Samples with marker call rates <95%, sample call rates <90%, or sex discordance were removed. Genotypes were phased (Eagle) and imputed to the 1000 Genomes reference panel (1000G Phase3) using the Michigan Imputation Server. In total, 39,296,776 polymorphic variants were imputed. Genetic ancestry was calculated from common, high quality SNPs (MAF > 0.05, missingness < 0.1) by training a kernel density estimator on principal components (PCs) from HapMap3 samples, determining the likelihood of each individual being from that ancestral superclass, and assigning the ancestral superclass based on the likelihoods. Individuals assigned to two ancestral superclasses were preferentially assigned to the non-European superclass.[2]

## Phenotype Ascertainment

For individual level analyses in Penn Medicine Biobank, phenotype prevalence was determined by querying the electronic health record. International Classification of Diseases (ICD) 9/10 and Current Procedural Terminology (CPT) codes, in addition to laboratory measurements and vital signs, were used to identify height, weight, BMI, smoking status, diagnoses of heart failure, hypertension, diabetes mellitus, chronic kidney disease, sleep apnea, stroke, thyroid disease, valvular heart disease, and cardiac surgery. Clinical phenotypes could be ascertained for 6548 individuals with high-quality genotype data. Data was extracted as of January 2017. Unless otherwise specified, individuals were defined as cases when having 2 or more relevant ICD9/10 codes, and for procedural diagnoses, 1 or more CPT codes. Individuals not meeting case criteria were considered controls for each diagnosis. Atrial fibrillation was defined using ICD9/10 codes: 427.31, I48.0, I48.1, I48.2, I48.91. Hypertension was defined using ICD9/10 codes: 401, 402, 403, 404, 405, 437.2, 642.0, 642.1, 642.2, I10, I11, I12, I13, I14, I15, I16, I67.4, O10. Diabetes was defined as either 2 or more ICD9 (250*, 357*, 362, 366.41, 648) or ICD10 (E10*, E11*, E13*, O24*) AND a prescription for insulin, an oral diabetes medication, or statin; 2 or more ICD9 or ICD10 codes AND either a random glucose >200, fasting glucose >125, or hemoglobin A1c test ordered; A prescription for insulin, an oral diabetes medication, or statin, AND an abnormal glucose lab result. Chronic Kidney disease was defined using ICD9/10 codes: 585*, 403*, 250.4, N18*, I12*, E10.2, E11.2, and eGFR <= 60. Heart Failure was defined using ICD9/10 codes: 428*, I50*. Stroke was defined using ICD9/10 codes: 433*, 434*, 435*, V12.54, I63*, G45*, Z86.73. Thyroid disease was defined using ICD9/10 codes: 242*, 242.1*, 242.2*, 242.3*, 242.4*, 242.8*, 242.9*, E05.30, E05.31, E05.80, E05.81, E05.00, E05.20, E05.10, E05.11, E05.21, E05.01, E05.91. Valvular heart disease was defined using ICD9/10 codes: 394, 394.1, 394.2, 394.9, 395, 395.1, 395.2, 395.9, 396, 396.1, 396.2, 396.3, 396.8, 396.9, 397, 397.1, 397.9, 424, 424.1, 424.2, 424.3, V42.2, V43.3, Z95.1, 746.4, 745.81, 746.09, Q23.0, Q23.1, Q23.3, Q23.8, Q23.9, Q22.2, Q24.4, IO5*, IO6*, IO7*, IO8*, IO9.1, I34*, I35*, I36*, I37*, I38*. Sleep apnea was defined using PheCodes (version 1.2 ICD10-CM) 327.3, 327.31, 327.32. Cardiac surgery was defined using ICD9/10 codes: 35*, 36*, 37* and CPT codes: 00560, 00561, 00562, 00563, 00566, 00567, 32160, 33015, 33020, 33025, 33030, 33031, 33031, 33050, 33120, 33130, 33141, 33246, 33250, 33251, 33253, 33257, 33257, 33258, 33259, 33261, 33300, 33305, 33310, 33315, 33321, 33322, 33330, 33332, 33335, 33361, 33362, 33363, 33364, 33365, 33366, 33367, 33368, 33369, 33400, 33401, 33403, 33404, 33405, 33406, 33406, 33410, 33411, 33412, 33413, 33415, 33416, 33417, 33420, 33422, 33425, 33426, 33427, 33430, 33430, 33460, 33463, 33464, 33465, 33468, 33470, 33471, 33472, 33474, 33475, 33476, 33478, 33496, 33500, 33501, 33502, 33503, 33504, 33505, 33506, 33507, 33510, 33511, 33512, 33513, 33514, 33516, 33517, 33518, 33519, 33521, 33522, 33523, 33530, 33533, 33534, 33535, 33536, 33542, 33545, 33545, 33572, 33600, 33602, 33606, 33608, 33610, 33611, 33612, 33615, 33617, 33619, 33641, 33645, 33647, 33660, 33665, 33670, 33681, 33684, 33688, 33690, 33692, 33694, 33697, 33702, 33710, 33720, 33722, 33730, 33732, 33735, 33736, 33737, 33750, 33755, 33762, 33764, 33766, 33767, 33770, 33771, 33774, 33775, 33776, 33777, 33778, 33779, 33780, 33781, 33786, 33788, 33800, 33813, 33814, 33820, 33822, 33824, 33840, 33845, 33851, 33852, 33853, 33860, 33861, 33863, 33864, 33870, 33875, 33877, 33891, 33910, 33915, 33916, 33917, 33918, 33919, 33920, 33922, 33924, 33935, 33945, 33967, 33968, 33970, 33971, 33973, 33974, 33975, 33976, 33977, 33978, 33999, 1021150, 4110F. For individuals with available echocardiogram data, left atrial size measured in the parasternal long axis view was extracted. For individuals with more than one measure available the median left atrial diameter value was used.

# Supplemental References

1. Burgess S, Davies NM, Thompson SG. Bias due to participant overlap in two-sample Mendelian randomization. Genet Epidemiol. 2016;40: 597–608. doi:10.1002/gepi.21998

2. Dewey FE, Murray MF, Overton JD, Habegger L, Leader JB, Fetterolf SN, et al. Distribution and clinical impact of functional variants in 50,726 whole-exome sequences from the DiscovEHR study. Science (80- ). 2016. doi:10.1126/science.aaf6814

3. Chang CC, Chow CC, Tellier LCAM, Vattikuti S, Purcell SM, Lee JJ. Second-generation PLINK: Rising to the challenge of larger and richer datasets. Gigascience. 2015;4. doi:10.1186/s13742-015-0047-8
