# Supplementary material for: Genetics of height and risk of atrial fibrillation: A Mendelian randomization study
Source: PLoS Med. 2020 Oct 8;17(10):e1003288. doi: 10.1371/journal.pmed.1003288 (PMC7544133; doi:10.1371/journal.pmed.1003288)
Supplement: Attachment — Submitted filename: Afib-Height_response-to-reviewers_20200213.docx [file pmed.1003288.s015.docx]

**Requests from the editors:**

1. **Comment:** *Article meta-data- some of the meta-data are incomplete and we request your attention to providing this information for instance- funding information, ethics statement, competing interests etc. Please contact* [*plosmedicine@plos.org*](mailto:plosmedicine@plos.org) *if you need assistance*

**Response:** We have updated the meta-data for the manuscript on the submission website.

2. **Comment:** *Abstract- Please structure your abstract using the PLOS Medicine headings (Background, Methods* and *Findings, Conclusions).*

*Abstract Background: Provide the context of why the study is important. The final sentence should clearly state the study question.*

**Response:** We have reformatted the abstract to the PLOS Medicine format.

**Manuscript Changes:** Abstract (Page 2, lines 40-60)

“**Background:** Observational studies have identified height as a strong risk factor for atrial fibrillation (AF), but this finding may be limited by residual confounding. We aimed to leverage genetic variation in height within the Mendelian randomization framework to determine whether height has a causal effect on risk of atrial fibrillation.

**Methods and Findings:** In summary-level analyses, Mendelian randomization (MR) was performed using summary statistics from genome-wide association studies of height (GIANT; 693,529 individuals) and atrial fibrillation (AFGen; 65,446 cases and 522,744 controls), finding that each 1 standard deviation increase in genetically-predicted height increased odds of AF (Odds ratio [OR] 1.34; 95% Confidence Interval [CI] 1.29 to 1.40; p = 5x10^-42^). These results remained consistent in sensitivity analyses with MR methods that make different assumptions about the presence of pleiotropy, and when accounting for the effects of traditional cardiovascular risk factors on AF. Individual-level phenome-wide association studies of height and a height genetic risk score were performed among 6567 European-ancestry participants of the Penn Medicine Biobank (median age at enrollment 63 years, interquartile range 55-72), confirming prior observational associations between height and AF. Individual-level MR confirmed each 1 standard deviation increase in height increased odds of AF, including adjustment for clinical and echocardiographic AF risk factors (OR 1.89; 95% CI 1.50 to 2.40; p = 0.007). The main limitations of this study may include potential bias from pleiotropic effects of genetic variants, and generalizability of individual-level findings to non-European populations.

**Conclusions:** Height is likely a positive causal risk factor for atrial fibrillation.”

3. **Comment:** *Abstract Methods and Findings:
   * Please ensure that all numbers presented in the abstract are present and identical to numbers presented in the main manuscript text.
   * Please include the study design, population and setting, number of participants, years during which the study took place, length of follow up, and main outcome measures.
   * Please quantify the main results (with 95% CIs and p values).
   * Please include the important dependent variables that are adjusted for in the analyses.
   * Please include the actual amounts and/or absolute risk(s) of relevant outcomes (including NNT or NNH where appropriate), not just relative risks or correlation coefficients. (example for absolute risks: PMID: 28399126). 
   * In the last sentence of the Abstract Methods and Findings section, please describe the main limitation(s) of the study's methodology.*

**Response:** We have reformatted the abstract to include these recommendations.

**Manuscript Changes:** Abstract (Page 2, lines 40-60)

4. **Comment:** *Abstract-“as well as incorporating height into population screening strategies for atrial fibrillation” I’m not sure how screening strategies fit into your study so I would remove this from the  conclusions*

**Response:** We have now removed this sentence from the abstract.

**Manuscript Changes:** Abstract (Page 2, lines 40-60)

5. **Comment:** *Author summary : At this stage, we ask that you include a short, non-technical Author Summary of your research to make findings accessible to a wide audience that includes both scientists and non-scientists. The Author Summary should immediately follow the Abstract in your revised manuscript. This text is subject to editorial change and should be distinct from the scientific abstract. Please see our author guidelines for more information:* [*https://journals.plos.org/plosmedicine/s/revising-your-manuscript#loc-author-summary*](https://journals.plos.org/plosmedicine/s/revising-your-manuscript#loc-author-summary)

**Response:** We have now included an author summary.

**Manuscript changes:** Author Summary (Page 3, lines 62-80)

**AUTHOR SUMMARY**

**Why was this study done?**

- Studies have identified height as a risk factor for atrial fibrillation, a common abnormal heart rhythm.
- Whether being taller actually causes atrial fibrillation, or if this association is an artifact of prior study designs remains unclear.

**What did the researchers do and find?**

- We leveraged randomly-allocated genetic variants associated with height within the Mendelian randomization framework to study the effects of height on risk of atrial fibrillation.
- Genetic variants associated with taller stature increased risk of atrial fibrillation. This finding was consistent across multiple analysis methods, including when accounting for other known atrial fibrillation risk factors.

**What do these findings mean?**

- Taller individuals are likely to be at increased risk of atrial fibrillation.
- Future research is needed to better define the pathways connecting height to atrial fibrillation.

6. **Comment:** *References- please provide a full stop after the brackets, for example [1*].

**Response:** We have now updated the manuscript with reformatted references that include a full stop following brackets.

7. **Comment:** *Please provide 95% CIs and p values throughout and use standard notation such as p>0.001 or p=xxx if over 0.001*

**Response:** P-value and 95% confidence intervals are included throughout the manuscript. For genetic analyses, we have included the quantitative p-values (rather than discretizing values less than 0.001) to remain consistent with prior reporting of Mendelian randomization and genetic analyses (including those published in PLOS Medicine; see <https://doi.org/10.1371/journal.pmed.1002833>). In the summary demographic tables (Table 1, Supplemental Tables 6-8), p-values are truncated at 0.001.

8. **Comment:** *The Data Availability Statement (DAS) requires revision. For each data source used in your study:
   a) If the data are freely or publicly available, note this and state the location of the data: within the paper, in Supporting Information files, or in a public repository (include the DOI or accession number).
   b) If the data are owned by a third party but freely available upon request, please note this and state the owner of the data set and contact information for data requests (web or email address). Note that a study author cannot be the contact person for the data.
    c) If the data are not freely available, please describe briefly the ethical, legal, or contractual restriction that prevents you from sharing it. Please also include an appropriate contact (web or email address) for inquiries (again, this cannot be a study author).*

**Response:** We have updated the Data Availability Statement to include information about the availability of data from the Penn Medicine BioBank, which are not publicly available due to their sensitive individual-level nature, but which may be made available with the appropriate ethical approval and data sharing agreements.

**Manuscript changes:** Data Availability Statement (pages 20-21, lines 455-464)

“GWAS summary statistics for height are publicly available, and can be downloaded from the GIANT consortium website (https://portals.broadinstitute.org/collaboration/giant/index.php/GIANT_consortium_data_files). Summary statistics for atrial fibrillation were contributed by the AFGen consortium (http://afgen.org), are publicly available, and may be downloaded from the Variant to Function Knowledge Portal (http://www.kp4cd.org/datasets/v2f). Individual-level data from the Penn Medicine BioBank are not publicly available due to their sensitive nature, however the data may be made available with the appropriate ethical approval and data sharing agreements (biobank@upenn.edu).”

9. **Comment:** *Please ensure that the study is reported according to the STROBE guideline, and include the completed STROBE checklist as Supporting Information. When completing the checklist, please use section and paragraph numbers, rather than page numbers. Please add the following statement, or similar, to the Methods: "This study is reported as per the Strengthening the Reporting of Observational Studies in Epidemiology STROBE guideline (S1 Checklist)."*

**Response:** We have now included the STROBE guideline checklist in the supplemental material.

**Manuscript Changes:** Materials and Methods (page 5, lines 117-118)

“This study is reported per the Strengthening the Reporting of Observational Studies in Epidemiology STROBE guideline (S1 Checklist).”

**Reviewer #1:**

1. **Comment:** *In the manuscript entitled " Genetics of Height and Risk of Atrial Fibrillation: A Mendelian Randomization Study " the authors investigated the association of height with incidence of atrial fibrillation.
   They performed a two-step approach with an initial analysis using summary-level data for GWAS of height and atrial fibrillation. In a second step they verified the results using individual-participant data from the Penn Medicine Biobank and found similar results. Even after adjusting for relevant clinical factors the results remained consistent. The authors conclude that height is causally associated with high risk of atrial fibrillation. The study size is adequate and the statistics used are appropriate. The manuscript is well written and the topic is of scientific relevance and interest. However, I have a few major concerns and comments:*

**Response:** We thank the reviewer for their careful read of our manuscript. We have worked to address the concerns outlined below.

2. **Comment:** *One concern is the missing link to the clinical relevance of the findings. How can these findings add to diagnosis, prevention or therapies in patients with AF in a clinically routine? e.g. a risk score? Should tall people try to lose weight? Can we try to compensate our stature? I think the manuscript would benefit from a section on this.*

**Response:** We have now included a section in the discussion commenting on the clinical relevance of these findings.

**Manuscript Changes:** Discussion (page 19, lines 410-421)

“The findings of this study have several clinical implications. Anthropometric characteristics have been included in clinical models that predict incident atrial fibrillation, including BMI in a model derived from the Framingham Heart Study, and height in the CHARGE-AF model from the Cohorts for Heart and Aging Research in Genomic Epidemiology Atrial Fibrillation Consortium [46–48]. While height is not a readily-modifiable risk factor, the recognition that taller individuals have increased risk of atrial fibrillation may prompt more aggressive management of modifiable cardiovascular risk factors like overweight/obesity, prediabetes/diabetes, hypertension, and alcohol/tobacco use in this subpopulation. Similarly, further mechanistic investigation of the height-atrial fibrillation link may identify novel modifiable pathways for intervention. Future study is needed to determine whether risk-prediction tools including height or other anthropometric factors can be used to improve screening and primary prevention of atrial fibrillation.”

3. **Comment:** *The finding that height and atrial fibrillation are causally related is interesting. Similar, fat-free mass has been described as an independent risk factor for atrial fibrillation (Tikkanen et al EHJ 2019). Regarding height and traits like fat-free mass, is there a genetic overlap? Do some of these variants affect both - height and fat-free mass?*

**Response:** Associations between other anthropometric risk factors with atrial fibrillation has been investigated in many prior studies, including the combined observational and Mendelian randomization analysis by Tikkanen et. al. investigating bioimpedance- and DEXA-derived measures of body composition (fat mass and fat-free mass). The reviewer raises an interesting question regarding overlap between height and other anthropometric measures. We used the PhenoScanner tool ([www.phenoscanner.medschl.cam.ac.uk](http://www.phenoscanner.medschl.cam.ac.uk)) to identify overlap between genetic variants associated with height and other traits (including both published GWAS and analyses of UK Biobank traits). Of the 707 variants included in the genetic instrument for height, 24 were associated (p < 5x10^-8^) with whole body fat mass (15 with negative relationship, 9 with positive relationship), and 165 with whole body fat-free mass (76 with negative relationship, 89 with positive relationship). Tikkanen et. al. found that greater fat and fat-free mass were associated with increased risk of atrial fibrillation, and concluded that “weight loss is likely to be a key factor in preventing AF, regardless of the person’s current body composition.” Because we focused our analyses on common, readily-obtainable clinical risk factors for atrial fibrillation, we did not include information on more advanced measures of body composition. We have added text to the discussion raising the possibility that the effects of height on body composition may influence the relationship between height and atrial fibrillation.

**Manuscript Changes:** Discussion (page 17, lines 383-388)

“Bioimpedance- and dual-energy X-ray absorptiometry-derived measures of body composition have also been associated with atrial fibrillation [44]. While the current study focused primarily on common cardiometabolic risk factors used in clinical practice, the possibility remains that more advanced anthropometric screening beyond height, weight, body mass index, and waist-hip ratio may explain some of the effect of increased height on atrial fibrillation.”

4. **Comment:** *Did the authors see any gender differences between men and women in their analyses?*

**Response:** In the two-sample Mendelian randomization analysis, sex-specific genome-wide association study summary statistics were not available, limiting our ability to perform stratified analysis. Nevertheless, the underlying summary statistics for the height genetic instrument were adjusted for sex, and the results from the two-sample analysis should be generalizable to men and women. At the individual level, we have now performed a sex-stratified analysis of the base-model adjusting for age and 6 genetic principal components, finding no substantial difference in the effect of height on atrial fibrillation between men and women (OR_Male_ = 1.65, 95% CI 1.39-1.97, p = 0.004; OR_Female_ = 1.97, 95% CI 1.56-2.49, p = 0.004), with overlapping 95% confidence intervals.

**Manuscript Changes:** Results (page 13, lines 334-336)

“Results were similar in sex-stratified analysis (OR_Male_ = 1.65 per 1-SD increase in height, 95% CI 1.39-1.97, p = 0.004; OR_Female_ = 1.97, 95% CI 1.56-2.49, p = 0.004).”

5. **Comment:** *If data on LA-size is available, why was this not included in the baseline table?*

**Response:** Left atrial size information by echocardiography was available for only a subset of participants. We have now included echo information in the baseline demographics table, and in new supplemental tables including baseline covariates across multiple strata of the Penn Medicine Biobank cohort, including those with complete phenotype information, and by height risk score quartile.

**Manuscript Changes:** Table 1, Supplemental tables 6-8

**Reviewer #2:**

1. **Comment:** *General comments
   Interesting and well conducted study that investigates the evidence for causality between height and incident atrial fibrillation. The authors state that their findings raises the possibility of investigating pathways of height. The authors also state that their findings suggest to use height in screening strategies for atrial fibrillation. That is not part of the research described in this paper, and seems overinterpretation of their results.*

**Response:** We agree with the comments from the reviewer (and editor) with regard to this conclusion, which overextends the results of the current study. We have removed suggestions for incorporating height into screening for atrial fibrillation. In reference to a comment from Reviewer #1, asking for additional clinical implications of these results, we have added a section to the discussion interpreting these findings in the context of previously developed risk scores that incorporate height and other anthropometric risk factors. Although clinical risk scores incorporating these anthropometric risk factors are predictive of incident atrial fibrillation, the clinical utility of these tools in screening and primary prevention remains unstudied.

**Manuscript Changes:** Discussion (page 19, lines 410-421)

“The findings of this study have several clinical implications. Anthropometric characteristics have been included in clinical models that predict incident atrial fibrillation, including BMI in a model derived from the Framingham Heart Study, and height in the CHARGE-AF model from the Cohorts for Heart and Aging Research in Genomic Epidemiology Atrial Fibrillation Consortium [46–48]. While height is not a readily-modifiable risk factor, the recognition that taller individuals have increased risk of atrial fibrillation may prompt more aggressive management of modifiable cardiovascular risk factors like overweight/obesity, prediabetes/diabetes, hypertension, and alcohol/tobacco use within this subpopulation. Similarly, further mechanistic investigation of the height-atrial fibrillation link may identify novel modifiable pathways for intervention. Future study is needed to determine whether risk-prediction tools including height or other anthropometric factors can be used to improve screening and primary prevention of atrial fibrillation.”

2. **Comment:** *Introduction
   The background and aim of the study are discussed. Clear overview of the manuscript is given.*

**Response:** We thank the reviewer for their comments – we have worked to address their suggestions outlined below.

3. **Comment:** *Materials and methods
   Methods are discussed in a well-arranged manner. According to the supplementary data atrial fibrillation is identified by ICD codes in the Penn dataset. However, atrial fibrillation ascertainment should be added in the materials and methods section of the paper itself.*

**Response:** We have now included the atrial fibrillation ascertainment to the main Materials and Methods section.

**Manuscript Changes:** Materials and Methods (pages 11-12, lines 255-258)

“Atrial fibrillation was defined using International Classification of Diseases (ICD) 9/10 codes: 427.31, I48.0, I48.1, I48.2, and I48.91. Atrial fibrillation ascertainment in the 2018 Roselli GWAS meta-analysis was study-specific, but could include diagnostic codes, electronic health record information, and self-report [25].”

4. **Comment:** *To accommodate the readers, please align the order of analyses in the methods and results section. In the results section the text starts with data of the individual-level analyses while the methods section starts with the summary-level two-sample MR.  The same applies to the discussion section where the analyses on population-level are discussed first; try to arrange a certain order to describe the results.*

**Response:** We have now re-aligned the presentation of the analyses in the Methods, Results, and Discussion section to focus first on the population-level analyses, followed by individual-level analyses.

**Manuscript Changes:** Materials and Methods, Results, Discussion

5. **Comment:** *Results
   In most literature about height 'centimeter' instead of 'inches' is used. If the authors chose to use centimeters instead of inches, the data is easier comparable with existing literature. It may be worth using the SI system or both to accommodate worldwide readers.*

**Response:** We have now updated the manuscript results to use the SI system.

6. **Comment:** *In the individual-level Mendelian randomization adjustment for 5 genetic principal components was chosen. Did the authors make a screen plot or was it an arbitrary estimate? Please provide a screen plot in supplementary data if possible.*

**Response:** We initially chose 5 principal components based on prior genetic analyses. At the suggestion of the reviewer, we have reviewed a scree plot of the variance explained by the principal components for European participants of the Penn Medicine Biobank. Based on this data, it appears that 6 principal components may explain slightly more variance than 5, and have repeated the individual-level analyses using 6 principal components. We have included the representative scree plot among European Penn Medicine Biobank participants for reference:

**Manuscript Changes:** Materials and Methods, Results, Figure 4, Supplemental Figure 3

7. **Comment:** *Discussion
   The authors also state that their findings suggest to use height in screening strategies for atrial fibrillation. That is not part of the research described in this paper, and seems overinterpretation of their results. Please remove.*

**Response:** We agree with the comments from the reviewer (and editor) with regard to this conclusion, which overextends the results of the current study. We have removed suggestions for incorporating height into screening for atrial fibrillation. In reference to a comment from Reviewer #1, asking for additional clinical implications of these results, we have added a section to the discussion interpreting these findings in the context of previously developed risk scores that incorporate height and other anthropometric risk factors. Although clinical risk scores incorporating these anthropometric risk factors are predictive of incident atrial fibrillation, the clinical utility of these tools in screening and primary prevention remains unstudied.

**Manuscript Changes:** Discussion (page 19, lines 410-421)

“The findings of this study have several clinical implications. Anthropometric characteristics have been included in clinical models that predict incident atrial fibrillation, including BMI in a model derived from the Framingham Heart Study, and height in the CHARGE-AF model from the Cohorts for Heart and Aging Research in Genomic Epidemiology Atrial Fibrillation Consortium [46–48]. While height is not a readily-modifiable risk factor, the recognition that taller individuals have increased risk of atrial fibrillation may prompt more aggressive management of modifiable cardiovascular risk factors like overweight/obesity, prediabetes/diabetes, hypertension, and alcohol/tobacco use among this subpopulation. Similarly, further mechanistic investigation of the height-atrial fibrillation link may identify novel modifiable pathways for intervention. Future study is needed to determine whether risk-prediction tools including height or other anthropometric factors can be used to improve screening and primary prevention of atrial fibrillation.”

**Reviewer #3:**

1. **Comment:** *The main claim of the paper is that developing atrial fibrillation is causally related to height. The main result is from a two-sample mendelian randomisation model, with data from two large GWAS studies whose summary data is publicly available. A clear scientific background is given, along with a rationale for investigating the association between height and atrial fibrillation. Due to the implausibility of conducting a randomised controlled trial with height as an exposure, Mendelian Randomisation is a sensible approach to take to investigate this association.

   This claim is not novel, there was a large study that demonstrated a very similar relationship (Lai FY, Nath M, Hamby SE, et al. Adult height and risk of 50 diseases: a combined epidemiological and genetic analysis. BMC Med 2018;16:187. doi:10.1186/s12916-018-1175-7). The claims made by that paper are properly placed in the context of previous literature. That paper looked only at people of European ancestry and did not do the level of sensitivity analysis shown here. This paper gives strong evidence to support this earlier paper, while also showing some generalisability to non-European populations. It also uses a secondary analysis on individual level data from Penn Medicine Biobank to support the main result and investigate some potential mechanisms. 

   The prior study also found a multitude of other diseases associated with genetically determined height such as reduced CAD and increased hip fracture. This paper is interesting in that it didn't find any significant confirmation of those other results as part of its PheWAS, potentially due to a higher standard of correction for multiple testing being applied. Sadly, the authors do not go into much detail about which of these earlier results appear on the PheWAS at a lower threshold of significance or discuss whether they have any evidence to contradict the results of the earlier paper in these diseases.  

   The main result supports the causal claim in the abstract, and the number and consistency of the sensitivity analyses give reassurance as to the robustness of these results. I have some concerns about some of the secondary analyses.  I detail these below. The selection of the genetic variables is sensible and well-described; both in the primary analysis and the sensitivity analysis with a reduced genetic instrument. The choice of analyses to perform is comprehensive and well-chosen, and for the most part, reasonably well explained. The discussion of the limitations of this study was appropriate. The results are mostly correctly interpreted and compared sensibly with other relevant studies.  The conclusion of this study was appropriate. The potential for clinical relevance is limited, due to the impracticalities of intervening on height, but the potential for investigating novel pathways or screening strategies is present. 

   This study follows most of the relevant guidelines in MR-STROBE; there are some specific and easy to fix queries detailed below. The paper is reasonably well-organised and should be accessible to non-specialists.

   The underlying research question is an important one, but given the earlier paper, it is not clear that these results are a substantial advance on existing knowledge. The results will be of interest to policymakers and clinicians, particularly those with an interest in screening strategies for atrial fibrillation.

   This paper is well done, but I think it is held back from being outstanding by the combination of its overall similarity to an earlier study and the lower statistical rigour in the more novel parts. It would be improved by a higher focus on the individual level analysis and the potential mechanisms for the effect.*

**Response:** We thank the reviewer for their comments. We have worked to address their concerns below.

2. **Comment:** *Major Concerns
   I am not convinced by the statistical rigour of the secondary analysis on the individual level data. This is a shame, as the inclusion of this data and the opportunity it affords for investigation of the potential mediators and mechanisms of the effect under investigation is one of the more novel parts of this paper.*

**Response:** We appreciate the reviewer’s careful attention and detailed consideration of the methods we used for the individual level analysis. We have taken these suggestions to heart and implemented them in the revised paper. We feel this has significantly increased the rigor of these analyses and thank the reviewer for their suggestions. Please see below for additional details and manuscript changes.

3. **Comment:** *The model is not sufficiently explained; I am somewhat confused as to what exactly has been done and what conclusions have been drawn from it. It would be helpful for the authors to expand this section, to clarify the hypothesis being tested and how they are interpreting the resulting odds ratios.*

**Response:** We have expanded our description of the individual level analysis to more fully explain the model. Our individual analysis was designed to test the hypothesis that increasing height is a causal risk factor for atrial fibrillation. Using individual level data with rich phenotypes from the Penn Medicine Biobank allowed us to adjust for important potential confounders that could not be considered in the two-sample summary-statistics analysis. This analysis also avoids some of the bias that could have been introduced in the two-sample analysis due to overlap between participants of the exposure and outcome studies as participants from the Penn Medicine Biobank are independent from the genome wide association study of height used to obtain the genetic instrument. Our models estimate the population-averaged effect of increased height on risk of atrial fibrillation using the two-stage method. The results of this analysis provide a causal estimate of the effect of increased height on risk of atrial fibrillation that serves as a complement to the population-level two-sample Mendelian randomization analysis. We have updated the “Individual-level Mendelian Randomization” subsection of the Materials and Methods section to better describe the analysis and interpretation.

**Manuscript Changes:** Materials and Methods (pages 9-10, lines 191-213)

“Individual-level MR was performed to complement the two-sample analysis. Many clinical risk factors for atrial fibrillation are not well-captured by genome-wide association studies (for example cardiac surgery and heart failure). These risk factors could therefore not be considered in the two-sample MR analysis using summary statistics. Further, because Penn Medicine Biobank participants were not included in the genome-wide association study of height, individual-level MR avoids potential bias from sample overlap in the population-level two-sample analysis using summary statistics. To consider these important risk factors, we estimated the population-averaged effect of increased height on risk of atrial fibrillation using the two-stage method [34]. This analysis included individuals with available height, genotype, and clinical covariate information.

The instrumental variable was a genetic risk score for height, computed from independent, genome-wide significant variants, weighted by effect on height in the 2018 GIANT + UKB GWAS meta-analysis. In the first stage of the two-stage process, a linear regression model was fitted with height as the dependent variable, and the genetic risk score for height as the independent variable, among the subset of individuals without atrial fibrillation. The model was then applied to the entire dataset, comprised of both atrial fibrillation cases and controls, to generate a fitted value for genetically-predicted height for all participants. In the second stage regression, a logistic regression model with robust standard errors was was used to test the association of fitted values of genetically-predicted height as the exposure, with atrial fibrillation as the outcome. In additional models, each stage was adjusted for clinical diagnoses and left atrial size to account for potential cofounders/mediators of the height-atrial fibrillation relationship. Sensitivity analysis was performed including only individuals who had available echocardiographic data.”

4. **Comment:** *[p9] "In the first stage of the two-stage process, a linear regression was conducted with standardized height as the dependent variable, and the
   standardized genetic risk score for height as the independent variable. In the second stage, a logistic regression model with robust standard errors was fit, incorporating both the residuals from the first stage and scaled height, with atrial fibrillation as the outcome."

   It is not clear whether the first stage was assessed only in controls, or if the cases were included as well. Including cases in this estimate could introduce bias to the model. I am unsure what scaled height refers to in the second stage but using an adjusted two-stage approach makes the odd ratio is very complex to interpret.* [*https://www.ncbi.nlm.nih.gov/pmc/articles/PMC5642006/*](https://www.ncbi.nlm.nih.gov/pmc/articles/PMC5642006/) *does not recommend adjustment on first-stage residuals, and it would be useful to the reader to have greater clarity on how conditioning the IV estimate on the residual should be interpreted as a result.*

**Response:** In the original analysis we performed the first stage model in all PMBB participants. We have now followed the reviewer’s recommendation to model the first stage among only participants without atrial fibrillation to minimize bias in the model. Similarly, we no longer use the control function estimator model (which conditions on the residual of the first stage regression), but rather the traditional two-stage model which incorporates a fitted value for height based on the first stage regression.

**Manuscript Changes:** Materials and Methods (pages 9-10, lines 203-212)

“In the first stage of the two-stage process, a linear regression model was fitted with height as the dependent variable, and the genetic risk score for height as the independent variable, among the subset of individuals without atrial fibrillation. The model was then applied to the entire dataset, comprised of both atrial fibrillation cases and controls, to generate a fitted value for genetically-predicted height for all participants. In the second stage regression, a logistic regression model with robust standard errors was was used to test the association of fitted values of genetically-predicted height as the exposure, with atrial fibrillation as the outcome. In additional models, each stage was adjusted for clinical diagnoses and left atrial size to account for potential cofounders/mediators of the height-atrial fibrillation relationship.”

5. **Comment:** *[p9] "In additional models, the second stage was adjusted for clinical diagnoses and left atrial size."
   This implies that the first stage was not adjusted for these covariates. In addition to the concerns above, this raises some questions over what the ORs in these additional models are representing when the second stage, but not the first is adjusted for covariates. Could the authors please clarify what hypotheses are being tested here? A better alternative would be to use the same covariates in the first- and second-stage IV regressions. If a specific conditional estimate is intended, then it needs much more explanation to the reader. See* [*http://www.phpc.cam.ac.uk/ceu/files/2012/11/letterthirdone010611-1.pdf*](http://www.phpc.cam.ac.uk/ceu/files/2012/11/letterthirdone010611-1.pdf) *&* [*https://www.bmj.com/content/362/bmj.k601*](https://www.bmj.com/content/362/bmj.k601) *for further detail on this issue.*

**Response:** We now recognize the potential for bias that may have been introduced by adjusting only the second stage regressions for clinical covariates and left atrial size. In the sequential models we now adjust both the first and second stage regressions for the same covariates in order to obtain population-averaged estimates of the effect of increased height on atrial fibrillation, rather than conditional estimates.

**Manuscript Changes:** Materials and Methods (pages 9-10, lines 203-212)

“In the first stage of the two-stage process, a linear regression model was fitted with height as the dependent variable, and the genetic risk score for height as the independent variable, among the subset of individuals without atrial fibrillation. The model was then applied to the entire dataset, comprised of both atrial fibrillation cases and controls, to generate a fitted value for genetically-predicted height for all participants. In the second stage regression, a logistic regression model with robust standard errors was was used to test the association of fitted values of genetically-predicted height as the exposure, with atrial fibrillation as the outcome. In additional models, each stage was adjusted for clinical diagnoses and left atrial size to account for potential cofounders/mediators of the height-atrial fibrillation relationship.”

6. **Comment:** *It is also worth noting that the samples used for this MR analysis were also used in determining the instrument (as part of the "Multi-ethnic genome-wide association study for atrial fibrillation")*

**Response:** A small number of participants from Penn were included in the AFGen Multi-ethnic genome-wide association study for atrial fibrillation (732 atrial fibrillation cases and 379 controls), though the AFGen study was used only as the outcome (and not the instrument) of the two-sample Mendelian randomization analysis rather than the individual-level analysis. The GIANT + UKB meta-analysis of height which was used to derive the height genetic instrument for both the two-sample and individual-level analyses also included a small number of individuals recruited from the University of Pennsylvania through the PennCATH study and MIGEN Consortium, although recruitment for this study occurred separately from the Penn Medicine Biobank.

7. **Comment:** *[P15] "Using individual-level MR analysis, we found that the relationship between height and atrial fibrillation appears to be independent of traditional clinical and echocardiographic risk factors for atrial fibrillation."

   This is too strong a statement. I am unconvinced that this claim can be justified from the paper as written. Either the authors need to expand and explain the results that have led to this, or they need to soften this conclusion.*

**Response:** We have softened this claim in the conclusion of the manuscript. We performed extensive multivariable modeling at both the population and individual levels, adjusting for traditional atrial fibrillation risk factors. In these analyses, we found height remained a strong causal risk factor, without substantial attenuation of the effect estimate despite adjustment for traditional atrial fibrillation risk factors. While it is possible (perhaps even likely) that the effect of height is mediated through another measurable risk factor, we did not find evidence to suggest this effect is attenuated by traditional atrial fibrillation risk factors. Overall, we provide strong evidence across the both population-level and individual-level analyses that consistently identify height as a strong, independent causal risk factor for atrial fibrillation.

**Manuscript Changes:** Discussion (page 16, lines 352-354)

“Mendelian randomization analysis at both the population and individual levels suggested height remains a causal risk factor for atrial fibrillation even after adjustment for other traditional risk factors.”

8. **Comment:** *Minor Concerns
   MR Assumptions:
   There is no discussion of the underlying MR assumptions & how they are justified. While some tests of the assumptions have been performed (e.g. MR-Egger to check for heterogeneity), these tests aren't explicitly linked to the underlying MR assumptions. It is critical for these assumptions & the evidence for them to be explicit so that the reader can use them to evaluate the validity of the model.*

**Response:** We have now included a section within the Materials and Methods section titled “Mendelian Randomization Assumptions.”

**Manuscript Changes:** Materials and Methods (pages 10-11, lines 216-235)

“Mendelian randomization relies on the naturally random assortment of genetic variants at conception to provide unbiased estimates of the effect of an exposure (in this case height) on an outcome (atrial fibrillation). To provide unbiased estimates, Mendelian randomization depends on three key assumptions [35]. First, genetic instruments must be associated with the exposure of interest (relevance assumption). Weak instruments that are not strongly associated with the exposure of interest may result in biased estimates. To estimate the strength of the height genetic instrument, the F-statistic was calculated **(Supplemental Table 1)**. The second assumption is that no confounders of the SNP-outcome association are present. This assumption is not readily testable because some confounders may not be measured, although at both the two-sample and individual-level the MR analyses were adjusted for common cardiovascular risk factors. Further, because genetic variants are randomly allocated at conception, confounders of the SNP-outcome association should be distributed across the population. The third assumption is that the effect of the genetic instrument on atrial fibrillation is entirely through the effect on height (exclusion restriction). We leveraged multiple MR methods including weighted median, MR-Egger, and MR-PRESSO, which are more robust to the presence of horizontal pleiotropy and potential violations of the exclusion restriction assumption [26]. Finally, Mendelian randomization assumes that genetically-mediated effects are similar to exogenous effects. In this case, height is highly heritable, although the effect of changes in stature related to environmental exposures (eg. fetal growth restriction, malnutrition) may differ from the genetic effects assessed here.”

9. **Comment:** *Information on the data sources for the two-sample MR:

   The first line of the methods section says "Summary-level data for GWAS of height and atrial fibrillation were obtained." but it requires some effort from the reader to discover exactly where this data comes from. Based on the references in the previous section, the AF associations comes from the Nature paper "Multi-ethnic genome-wide association study for atrial fibrillation", which has data from UK Biobank, Biobank Japan, & others - including Penn Medicine Biobank. The height associations come from "Meta-analysis of genome-wide association studies for height and body mass index in ∼700000 individuals of European ancestry", which shows that the data for height comes from a meta-analysis of data from UK Biobank & Wood et. al (which takes data from 79 smaller studies).

   Firstly, the reader should not have to read 3-4 references to determine the underlying population of the study. Please provide a clear account of the source of the data, it's underlying population etc. This is particularly relevant as the multi-ethnicity of the populations is one of the strengths of the study. Please include results on the heterogeneity of the different populations of your various data sources - the table for the Penn Medicine Biobank is excellent and it would be helpful to have as similar as possible for the populations that the AF & height data came from.  A table in on the individual level data comparing the 25th and 75th percentile of your genetic risk score for the Penn Medicine Biobank data could also provide useful context for considering the multivariate genetic effects.*

**Response:** We have now updated the Materials and Methods section to more specifically detail the generation of the height genetic instrument, and better describe the 2018 meta-analysis by Yengo et. al. from which this data was derived. We have similarly described the 2018 study by Roselli et. al. from which the atrial fibrillation outcome data was derived. We agree with the reviewer that baseline characteristics from these populations would be of interest to the reader. Unfortunately, the Yengo et. al. meta-analysis does not provide descriptive statistics in the main publication or supplemental materials. Limited baseline characteristics (age and anthropometric measures) from the prior 2014 GIANT GWAS of height by Locke et. al. are available in the supplemental materials of that publication, however this analysis represents less than half of the 2018 study. Similarly, only limited baseline characteristics from the 2018 Roselli atrial fibrillation GWAS are available in the supplemental materials of that publication.

For the individual-level data, we have added a supplemental table including summary demographics stratified by quartile of the height genetic risk score.

**Manuscript Changes:** Materials and Methods (pages 5-6, lines 121-137)

“Summary-level data for height was obtained from a 2018 meta-analysis of genome wide association studies of height [23]. This analysis was a fixed-effects meta-analysis combining results from a genome-wide association of height performed among 456,426 participants from the UK Biobank (adjusted for age, sex, recruitment center, genotyping batch, and 10 genetic principal components), and results from a 2014 genome-wide association study published by the GIANT (Genetic Investigation of ANthropometric Traits) Consortium, which included 253,288 participants from 79 studies (adjusted for age, sex, and study-specific covariates). Genetic variants associated with height at genome-wide significance (p < 5×10^-8^) were then LD-pruned (distance threshold = 10,000kb, r^2^ = 0.001) using the *clump_data* command in the *TwoSampleMR* package in R to identifiy an independent set of variants to serve as a genetic instrument for height [24]. The independent variants associated with height at genome-wide significance (p < 5×10^-8^), SNPs were then harmonized with variants from the 2018 Roselli et al. atrial fibrillation GWAS from the AFGen (Atrial Fibrillation Genetics Consortium). This study included 65,446 atrial fibrillation cases and 522,744 controls from more than 50 studies (84.2% European, 12.5% Japanese, 2% African American, and 1.3% Brazilian and Hispanic), including participants UK Biobank, Biobank Japan, other international biobanks, and international cardiovascular cohort studies (adjusted for age, sex, and study-specific covariates) [25].”

Supplemental Table 7:

| **Characteristic** | **Statistic** | **Height PRS Quartile** | | | | **p-value** |
| --- | --- | --- | --- | --- | --- | --- |
|  |  | **1, N = 1642** | **2, N = 1642** | **3, N = 1642** | **4, N = 1641** |  |
| Age | mean (SD) | 63 (13) | 62 (14) | 62 (14) | 62 (14) | 0.032 |
| Sex (Female) | n (%) | 620 (38%) | 618 (38%) | 654 (40%) | 615 (37%) | 0.5 |
| BMI | mean (SD) | 29 (6) | 29 (7) | 29 (6) | 29 (6) | 0.008 |
| Height (cm) | mean (SD) | 169 (11) | 171 (11) | 172 (11) | 175 (10) | <0.001 |
| Weight (kg) | mean (SD) | 82 (21) | 86 (21) | 85 (21) | 89 (23) | <0.001 |
| Atrial Fibrillation | n (%) | 730 (44%) | 728 (44%) | 750 (46%) | 821 (50%) | 0.003 |
| Hypertension | n (%) | 979 (60%) | 967 (59%) | 995 (61%) | 975 (59%) | 0.8 |
| Coronary Artery Disease | n (%) | 920 (56%) | 873 (53%) | 864 (53%) | 854 (52%) | 0.1 |
| Heart Failure | n (%) | 698 (43%) | 644 (39%) | 648 (39%) | 671 (41%) | 0.2 |
| Hyperlipidemia | n (%) | 967 (59%) | 944 (57%) | 952 (58%) | 912 (56%) | 0.3 |
| Diabetes | n (%) | 276 (17%) | 253 (15%) | 292 (18%) | 272 (17%) | 0.3 |
| Chronic Kidney Disease | n (%) | 272 (17%) | 219 (13%) | 254 (15%) | 232 (14%) | 0.047 |
| Sleep apnea | n (%) | 255 (16%) | 262 (16%) | 264 (16%) | 264 (16%) | >0.9 |
| Stroke | n (%) | 347 (21%) | 327 (20%) | 313 (19%) | 312 (19%) | 0.4 |
| Thyroid Disease | n (%) | 37 (2.3%) | 31 (1.9%) | 37 (2.3%) | 44 (2.7%) | 0.5 |
| Cardiac Surgery | n (%) | 714 (43%) | 703 (43%) | 702 (43%) | 774 (47%) | 0.032 |
| Valve Disease | n (%) | 605 (37%) | 564 (34%) | 584 (36%) | 556 (34%) | 0.3 |
| Smoking (ever) | n (%) | 814 (50%) | 809 (49%) | 789 (48%) | 768 (47%) | 0.4 |
| Left atrial diameter (cm) | mean (SD) | 4.20 (0.84) | 4.16 (0.81) | 4.17 (0.81) | 4.28 (0.82) | 0.033 |

10. **Comment:** *Also, UK Biobank's European participants are included in both studies, giving an overlap of at least 50%. This is briefly mentioned in the limitations section of the discussion, but a clear statement as to the level of the overlap & its estimate on the impact of this overlap should be given.*  [*https://sb452.shinyapps.io/overlap/*](https://sb452.shinyapps.io/overlap/) *may be useful here.*

**Response:** We have now estimated the effect of possible sample overlap using the above calculator. Because of the strong instruments for height and overall large sample size for both the height and atrial fibrillation genome-wide association studies, the effect of sample overlap was estimated to be negligible across the entire range of possible overlap (0 to 100%). We have added a section to the Supplemental Methods describing this estimation, and a table including bias estimates for different levels of sample overlap.

**Manuscript Changes:** Materials and Methods (page 7, lines 151-153)

“Bias due to sample overlap between the exposure and outcome genome-wide association studies was estimated and found to be negligible (**Supplemental Methods**) [32].”

Supplemental Methods

The results of Mendelian randomization analyses may be biased, although the effects may differ depending upon the underlying analysis (single- vs. two-sample), type of outcome (binary vs. continuous), and sample overlap. The effects of weak instrument bias are well-characterized in the analysis by Burgess, Davies, and Thompson [1]. Weak instrument bias in the single-sample setting tends to bias estimates toward the observational estimates, while in the two-sample setting estimates are biased towards the null. With a binary outcome, estimating the instrument-exposure relationship in control participants only results in negligible bias, regardless of sample overlap. When the instrument-exposure association is estimated in both cases and controls, however, substantial bias may result. Two-sample MR often relies on effect estimates derived from large consortia, with instrument-exposure associations estimated in both cases and controls for the outcome of interest. Sample overlap may therefore lead to bias in the MR estimates, even in the case of two-sample MR. In the current study, a substantial proportion of UK Biobank participants were included in the genome-wide association studies of height and atrial fibrillation, which could potentially lead to biased estimates of the effect of height and atrial fibrillation. To estimate the potential bias due to varying proportions sample overlap, we used the calculator available at <https://sb452.shinyapps.io/overlap/>. For a range of sample overlaps (0 to 100%) we estimated the potential bias of the 707-variant genetic instrument explaining 11.2% of the variation in height, among 693,529 individuals in the GIANT + UKB height GWAS, and 588,190 individuals in the Roselli et. al. 2018 AFGen atrial fibrillation GWAS, with case-proportion of 0.11, and observational log-odds estimate of the effect of 1 standard deviation increase in height on risk of atrial fibrillation of 0.408 based on the observational analysis in the Penn Medicine Biobank. Across the entire range of possible sample overlap we detected no substantial bias. For reference, the GIANT + UKB GWAS of height included 456,426 participant from the UK Biobank, while the AFGen GWAS of atrial fibrillation included 351,017. UK Biobank participants.

| **Overlap Proportion** | **Bias** | **Type I error rate** |
| --- | --- | --- |
| 0 | 0 | 0.05 |
| 0.1 | 0 | 0.05 |
| 0.2 | 0.001 | 0.05 |
| 0.3 | 0.001 | 0.05 |
| 0.4 | 0.001 | 0.05 |
| 0.5 | 0.002 | 0.05 |
| 0.6 | 0.002 | 0.05 |
| 0.7 | 0.002 | 0.05 |
| 0.8 | 0.003 | 0.06 |
| 0.9 | 0.003 | 0.06 |
| 1 | 0.003 | 0.06 |

11. **Comment:** *There is no comment on how the summary statistics were calculated for each data source - were similar covariates included in both calculations?*

**Response:** Both the height and atrial fibrillation GWAS studies were adjusted for age, sex, and study-specific covariates. As with the demographic information, these covariates are not readily specified in the main analyses or supplemental materials. We have updated the Materials and Methods section (as above), with information about the height and atrial fibrillation studies used as the main exposure and outcome in our analysis.

**Manuscript Changes:** Materials and Methods (pages 5-6, lines 121-137)

“Summary-level data for height was obtained from a 2018 meta-analysis of genome wide association studies of height [23]. This analysis was a fixed-effects meta-analysis combining results from a genome-wide association of height performed among 456,426 participants from the UK Biobank (adjusted for age, sex, recruitment center, genotyping batch, and 10 genetic principal components), and results from a 2014 genome-wide association study published by the GIANT (Genetic Investigation of ANthropometric Traits) Consortium, which included 253,288 participants from 79 studies (adjusted for age, sex, and study-specific covariates). Genetic variants associated with height at genome-wide significance (p < 5×10^-8^) were then LD-pruned (distance threshold = 10,000kb, r^2^ = 0.001) using the *clump_data* command in the *TwoSampleMR* package in R to identifiy an independent set of variants to serve as a genetic instrument for height [24]. The independent variants associated with height at genome-wide significance (p < 5×10^-8^), SNPs were then harmonized with variants from the 2018 Roselli et al. atrial fibrillation GWAS from the AFGen (Atrial Fibrillation Genetics Consortium). This study included 65,446 atrial fibrillation cases and 522,744 controls from more than 50 studies (84.2% European, 12.5% Japanese, 2% African American, and 1.3% Brazilian and Hispanic), including participants UK Biobank, Biobank Japan, other international biobanks, and international cardiovascular cohort studies (adjusted for age, sex, and study-specific covariates) [25].”

12. **Comment:** *Multiple definitions of AF
    Different definitions of AF are used by different data sources. e.g. from "Multi-ethnic genome-wide study...": "Ascertainment of AF in the UKBB includes samples with one or more of the following codes: non-cancer illness code, self-reported (1471, 1483); operation code (1524); diagnoses - main/secondary ICD10 (I48, I48.0-4, I48.9); underlying (primary/secondary) cause of death: ICD10 (I48, I48.0-4, I48.9); diagnoses - main/secondary ICD9 (4273); operative procedures - main/secondary OPCS (K57.1, K62.1-4)" whereas the definition used in the Penn Medicine Biobank definition is  "Atrial fibrillation was defined using ICD9/10 codes: 427.31, I48.0, I48.1, I48.2, I48.91.". I do not think this is problematic, but it should be made clear to the reader.*

**Response:** We have now updated the main Phenotype Ascertainment section of the Materials and Methods section to include ICD9/10 codes used to define atrial fibrillation within the Penn Medicine Biobank. We have also made note that the atrial fibrillation definitions varied among the contributors to the AFGen atrial fibrillation GWAS analysis.

**Manuscript Changes:** Materials and Methods (pages 11-12, lines 255-259)

“Atrial fibrillation was defined using International Classification of Diseases (ICD) 9/10 codes: 427.31, I48.0, I48.1, I48.2, and I48.91. Atrial fibrillation ascertainment in the 2018 Roselli GWAS meta-analysis was study-specific, but could include diagnostic codes, electronic health record information, and self-report [25].”

13. **Comment:** *Typos/Corrections
    In methods, it says "The instrumental variable was a standardized genetic risk score for height, computed from
    independent, genome-wide significant variants, weighted by the effect on height in the GIANT
    GWAS (see Height Genetic Risk Score above)." (P9) but the section on Height Genetic Risk does not appear until P11.*

**Response:** We have now re-arranged the Materials and Methods section and this oversight has been resolved.

**Manuscript Changes:** Materials and Methods

14. **Comment:** *Multiple testing was managed via Bonferroni correction in the PheWAS analysis, but this is only stated on the relevant figure. Please make it clear in the body of the text also.*

**Response:** We have now included a sentence in the pheWAS portion of the Methods section describing the procedure for multiple testing correction.

**Manuscript Changes:** Materials and Methods (page 8, lines 187-188)

“The Bonferroni method was used to account for multiple testing (p = 0.05/1816 phenotypes).”

15. **Comment:** *Figure 3 is a little unclear.  I think it is plotting the OR of Height on AF but there is no scale provided (presumably per 1S.D. change in height?) from the Multivariable MR that also includes the variables named under covariate. It feels odd to describe the other risk factors includes in the multivariable MR as covariates - that implies more a measured confounder to my ears. The OR for the other risk factors should also be reported (some are in ST4, but not for the combined model)*

**Response:** We have now included a scale in the legend for this figure (now figure 2 after rearrangement of the results section), which describes the effect of height on atrial fibrillation (per 1-SD change in height). We have renamed the covariate column to “model” to better reflect the multivariable nature of the analysis, and variables included in each multivariable model. We have also added an additional supplemental table reporting the effect estimates from the combined model.

**Manuscript Changes:** Figure 2

Multivariable Mendelian randomization was performed to jointly consider the effect of genetic variants for cardiometabolic traits and height on atrial fibrillation. The effect of a 1 standard deviation increase in height on risk of atrial fibrillation estimated by each model is displayed. The dashed line at OR 1.34 represents the effect of height on atrial fibrillation observed in the primary inverse variance weighted analysis. Odds Ratios (OR) per 1 standard deviation increase in height, 95% Confidence Intervals (CI) and P-values for Mendelian randomization estimates are displayed.

Supplemental Table 5

16. **Comment:** *p13 says "Inverse variance weighted modelling identified a significant association between increasing height and atrial fibrillation (OR 1.34; 95% CI 1.29 to 1.40; p = 5x10-42)" could the authors first clarify what the scale of height is for this OR - I am presuming per S.D. increase as with the Phenome-wide association studies, but it would be useful to have it explicitly.  Could the authors also please translate this into a clinically understandable result - e.g. what is the increased risk for a patient who is 10cm taller.*

**Response:** We have now updated the text to explicitly refer to the scale of the effect estimate. The reviewer raises an interesting point regarding the clinical meaning of the result, and requests scaling the result to a more understandable value. The population distribution of height may vary substantially depending on sex and population of interest (see: <https://doi.org/10.7554/eLife.13410>; <http://www.ncdrisc.org/height-mean-distribution.html>; <https://www.cdc.gov/nchs/data/nhsr/nhsr122-508.pdf>). In assessing an individual’s absolute risk of atrial fibrillation based on their height, one must consider the individual’s place within the height distribution among similar individuals (for example white men in the United Kingdom, or African American women in the United States), and the base rate of atrial fibrillation within that population. The current presentation of the results (eg. per 1-SD increase in height) allows this risk assessment to be generalized across populations. Providing an arbitrary estimate per 10cm increase in height could in fact lead to substantial over- or under-estimation of atrial fibrillation risk among populations other than the specific population to which the estimate was scaled. Summary demographic data is unavailable for the height GWAS study, although consistent with a recently published study (<https://doi.org/10.1093/ije/dyaa009>) using this data we have now scaled the two-sample Mendelian randomization estimate based on the February 2020 UK Biobank population standard deviation of height (9.28 cm). We have scaled the individual-level estimate based on the standard deviation of height within the Penn Medicine Biobank Population (11 cm).

**Manuscript Changes:** Materials and Methods (page 7, lines 153-156),

“The estimates for the effect of height on atrial fibrillation are reported per 1-standard deviation (SD) increase in height. A scaled estimate per 10-cm increase in height has also been calculated for the main analysis based on the February 2020 UK Biobank population standard deviation of height (9.28 cm).”

Results (page 12 line 276-277)

“This corresponds to an odds ratio of 1.37 (95% CI 1.31 to 1.44) per 10-cm increase in height among UK Biobank participants.”

Results (page 15, lines 333-334)

“This corresponds to an odds ratio of 1.66 (95% CI 1.46 to 1.86) per 10-cm increase in height among Penn Medicine Biobank participants.”

17. **Comment:** *ST2-4: please include s.e. and p-value for each odd ratio, not just the 95% confidence interval.*

**Response:** The supplemental tables have now been updated to include both standard error and p-value.

**Manuscript Changes:** Supplemental Tables ST1-5
