# Supplementary material for: Genetics of height and risk of atrial fibrillation: A Mendelian randomization study
Source: PLoS Med. 2020 Oct 8;17(10):e1003288. doi: 10.1371/journal.pmed.1003288 (PMC7544133; doi:10.1371/journal.pmed.1003288)
Supplement: Attachment — Submitted filename: Afib-Height_response-to-reviewers_20200731.docx [file pmed.1003288.s016.docx]

**EDITORIAL REQUIREMENTS:**

1. **Comment:** Please rephrase the first sentence of the Abstract Conclusions to: “In this study, we observed evidence that height is a positive causal risk factor for atrial fibrillation.”

**Response:** This sentence has been rephrased

2. **Comment:** Thank you for noting in the manuscript that there was no pre-registered or published analysis plan. Please also make sure that the Methods section transparently describes when analyses were planned, and when/why any data-driven changes to analyses took place.

**Response:** We have now added an additional sentence to the Methods section describing the timing of the analyses

**Manuscript Changes:** Materials and Methods (Page 6, lines 122-123)

“All analyses were planned prior to study initiation.”

3. **Comment:** Thank you for adding the scree plot to the supplemental files. Please add a bit more detail in the figure legend. Additionally, please include some discussion of how the 10 genetic principal components were selected (your response to point 19) in the Methods section, referencing S4 figure.

**Response:** The figure legend has now been updated, and additional discussion has been added to the Methods.

**Manuscript Changes:** Figure legends (Page 32, lines 683-685)

“Genetic Principal Component Scree Plot for Penn Medicine Biobank. Proportion of variance explained for each genetic principal component among European-ancestry participants of Penn Medicine Biobank.”

Methods (Page 10, lines 196-200; Page 11, lines 213-215)

“The selection of 10 genetic principal components for inclusion in the pheWAS analysis was determined a priori, without investigation of the variation of the data. For the more detailed individual-level Mendelian randomization analyses, 6 genetic principal components were identified qualitatively using a scree plot as explaining the majority of variation in the data (S4 Figure).”

“Six genetic principal components were selected qualitatively using a scree plot as explaining the majority of variation in the data (S4 Figure).”

4. **Comment:** Please also reference all Supplemental tables and figures from their relevant sections in the main text.

**Response:** All Supplemental tables and Figures are now referenced from the main text.

5. **Comment:** Please incorporate your response to point 18 (regarding excluding SNPS) in the Methods section of the manuscript, in case readers wonder the same.

**Response:** This response has now been added to the methods section

**Manuscript Changes:** Methods (Page 14, lines 298-300)

“The selection of SNPs using a nominal p-value association of 0.05 was an arbitrarily chosen threshold to more liberally account for potentially pleiotropic effects of height-associated SNPs.”

6. **Comment:** Please incorporate your response to point 22 (regarding family history of AF) into the Discussion section, in case other readers have the same thought.

**Response:** Additional discussion has been added regarding family history of atrial fibrillation.

**Manuscript Changes:** Discussion (Page 20-21, lines 435-438)

“As both height and atrial fibrillation are heritable, it is possible that height might act as a surrogate for family history of atrial fibrillation when this information is not readily available. Further study may clarify the risks of incident atrial fibrillation attributable to height and family history.”
